# Protective effect of Huanglian Pingwei San on DSS-induced ulcerative colitis in mice through amelioration of the inflammatory response and oxidative stress

**DOI:** 10.3389/fphar.2024.1484532

**Published:** 2024-12-04

**Authors:** Gengting Dong, Xiaoyan Pang, Ximin Wang, Lin Peng, Qili Xiao, Shunan Guo, Weibo Dai

**Affiliations:** Pharmacology Laboratory, Zhongshan Hospital of Traditional Chinese Medicine, Zhongshan, China

**Keywords:** Huanglian Pingwei San, ulcerative colitis, intestinal barrier, inflammation, oxidative stress

## Abstract

**Introduction:**

Ulcerative colitis (UC) results in the breakdown of the mucosal barrier caused by persistent inflammation and oxidative stress. Huanglian Pingwei San (HLPWS) is a commonly prescribed traditional Chinese medicine for treating colitis, but the precise mechanism remains unclear. The aim of this study was to systematically investigate the protective effect of HLPWS on UC mice and to elucidate the underlying mechanisms involved.

**Materials:**

UC mouse model was established in C57BL/6 mice via 2.25% dextran sulfate sodium (DSS). The chemical composition of HLPWS was examined through UPLC/MS Q-TOF analysis. The efficacy of HLPWS in treating UC was assessed. A TUNEL assay was used to detect apoptotic cells. An ELISA was used to evaluate the levels of inflammatory cytokines in colon tissues and serum. The percentages of Treg and Th17 cells were measured via flow cytometry. The protein expression in the colonic tissue was validated via immunohistochemistry (IHC) and Western blotting.

**Results:**

HLPWS significantly improved UC symptoms and colon tissue histology in mice. The structure and function of the intestinal barrier were restored by HLPWS treatment, as shown by increased DAO content, reduced levels of FITC-dextran, and increased protein expression of ZO-1, occludin, claudin, and MUC2. HLPWS dose-dependently decreased the number of apoptotic cells by inhibiting P53, P21, P27, cleaved caspase 3, and p-H2AX expression. HLPWS also reduced abnormal oxidative stress by reducing Keap1 expression and increasing Nrf2 and HO-1 levels. Furthermore, HLPWS rebalanced the Treg/Th17 ratio to alleviated inflammatory reactions in UC mice.

**Conclusion:**

These findings suggest that HLPWS alleviated colonic intestinal barrier dysfunction in UC mice by reducing oxidative stress and restoring immune balance. This study underscores the potential therapeutic benefits of HLPWS and highlights its potential as a future pharmaceutical candidate for UC treatment.

## 1 Introduction

Ulcerative colitis (UC) is characterized by symptoms such as bloody diarrhea, abdominal cramping, and frequent recurrence due to inflammatory cell infiltration (Kobayashi et al., 2020). Recent studies have indicated a rapid increase in the incidence of UC in many countries (Le Berre et al., 2023). The exact pathogenesis of UC remains largely unknown, disruption of intestinal epithelial barrier (IEB) function has been identified as key factor (Du and Ha, 2020). Apical tight junction (TJ) proteins, consisting of transmembrane proteins (occludin and claudin) and perimembrane proteins (ZO-1), play crucial roles in maintaining paracellular permeability and regulating IEB function (Maria Chiara Marchesi et al., 2017). While oxidative stress is essential for normal cell function at basal levels, increased oxidative stress can lead to further deterioration of intestinal barrier integrity and epithelial cell death (Cao et al., 2018). Oxidative stress has been shown to increase the transcriptional activity of P53, where P53 directly regulates P21 transcription, and subsequently triggers apoptosis. Free radicals in the body is a significant factor in UC, as it can disrupt tight junctions and allow inflammatory factors to penetrate and damage the intestinal mucosa (Tian et al., 2017). Inflammatory factors can also further exacerbate the oxidative stress. The increased release of inflammatory cytokines can affect the balance of Th17/Tregs, and ultimately disrupt the balance of the intestinal immune microenvironment. During the acute phase of UC activity, proinflammatory cytokines can exacerbate oxidative stress, disrupting redox balance and worsening inflammation by activating redox-sensitive factors that ultimately contribute to IEB destruction (Yang et al., 2017). Currently, the therapeutic strategy for UC primarily involves colectomy, pharmacotherapies, and stem cell-based therapy (Liu et al., 2023). Current pharmacotherapies focus mainly on preventing further colon damage, are limited in repairing the damaged colon and negatively impact quality of life due to side effects (Yan et al., 2020). To achieve a cure for UC, new drugs or alternative remedies are needed.

In traditional Chinese medicine (TCM), the predominant patterns observed in patients with UC are damp heat in the large intestine, deficiency of yang in the spleen and kidney, deficiency of the spleen with liver depression, weakness in the spleen and stomach, and accumulation of dampness with spleen deficiency (Zhang et al., 2022). Therefore, the treatment of UC patients requires not only clearing heat and dampness but also harmonizing qi and blood to tonify the spleen and stomach (Yang Z. et al., 2022). TCM, known for its multiple components, targets, and pathways, has been used to treat enteritis for centuries. Huanglian Pingwei San (HLPWS), composed of *Atractylodes Lancea* DC., *Houpoea officinalis* (Rehder & E.H.Wilson) N.H.Xia & C.Y.Wu, *Citrus nobilis subf. Reticulata* (Blanco) M. Hiroe, *Glycyrrhiza uralensis* Fisch. ex DC., *Coptis chinensis* Franch., was first described in 1739 AD in the Prescriptions of The Golden Mirror of Medicine (Yi zong jin jian). HLPWS comprises several active components that can exert a more comprehensive therapeutic effect through synergistic interactions, targeting multiple pathological mechanisms of UC. Previous research has shown that the fundamental formula Ping Wei San alleviates the degree of DSS-induced colitis by decreasing the level of the inflammatory protein NLRP3 (Zhang et al., 2019). The main component of HLPWS, *C. chinensis* Franch., has been found to suppress inflammatory responses and modulate immune function in UC (Yang Y. et al., 2022). Therefore, we speculated that the HLPWS, a combination of *C. chinensis* Franch, would be more effective in relieving UC symptoms than the foundational formula Ping Wei San. However, the specific mechanism by which HLPWS alleviates UC remains unclear. This study was conducted to explore the anti-inflammatory and antioxidative effects of HLPWS on DSS-induced UC in mice and elucidate the underlying mechanisms involved.

## 2 Materials and methods

### 2.1 Composition and preparation of HLPWS

*The* HLPWS was composed of *Atractylodes lancea* DC. (Cangzhu, 30 g, C22403246), *H. officinalis* (Rehder & E.H. Wilson) N.H. Xia & C.Y. Wu (Houpo, 10 g, 2404096), *Citrus reticulata* Blanco (Chenpi, 10 g, 2404140), *G. uralensis* Fisch. ex DC (Gancao, 6 g, C22403327), *C. chinensis* Franch (Huanglian, 15 g, C22404307). All the raw medicinal plants were purchased from Zhongshan Hospital of Traditional Chinese Medicine (Zhongshan, China). The HLPWS materials were moistened and boiled for 2 h, after which the extract was collected. After that, the extraction mixture was filtered with a 0.22 μm filter and then stored at −20°C.

### 2.2 Materials and reagents

Dextran sulfate sodium (DSS, MW: 36–50 kDa, DW-40L508) was obtained from MP Biomedicals Inc. (California, United States). ELISA kits for IL-6, IL-1β, LPS, IL-18, ROS, MPO, DAO,IL-22, IL-23, and IL-17 were purchased from Tianjin Anoric Biotechnology Co., Ltd. (#385220531, #370220510, #261220728, #375220706, #653220614,#411220531,#252220531,#621220706, #378220706, #374220706, Tianjin, China). The MDA and GSH assay kits were purchased from Beyotime Biotechnology Co., Ltd. (#S0131S, #S0057S; Shanghai, China). SOD and CAT kits were purchased from Nanjing Jiancheng Bioengineering Institution (#A001-3, #A007-1-1; Nanjing, China).

Primary antibodies against MUC2 (DF8390), HO-1 (AF5393), P53 (AF0879), P21 (AF6290), p-H2AX (AF3187), P27 (AF6324), ZO1 (AF5145), Occludin (DF7504), and Claudin (AF0127) were obtained from Affinity Biosciences Ltd. (OH, United States). The primary antibody against Keap1 (D199574) was obtained from Sangon Biotech Ltd. (Shanghai, China), and HRP-IgG (7074P2) and cleaved caspase 3 (#9661) were obtained from Cell Signaling Technology, Inc. (Boston, United States). A primary antibody against Nrf2 (A0674) was obtained from ABclonal Biotechnology Co., Ltd. (Wuhan, China). β-Actin (GB15003) was obtained from Servicebio Biotechnology Co., Ltd. (Wuhan, China).

The FOXP3/Transcription Factor Staining Buffer Kit (IC001), FIX&PERM Kit (GAS008/2), PMA/Ionomycin Mixture (250X, CS1001), and BFA/Monensin Mixture (250X, CS1002) were obtained from Lianke Multi Sciences Biotechnology Co., Ltd. (Hanzhou, China). The APC-conjugated anti-mouse CD4 antibody (E-AB-F1097UE), PE-conjugated anti-mouse Foxp3 antibody (E-AB-F1238D), and FITC-conjugated anti-mouse CD25 antibody (E-AB-F1102UC) were obtained from Elabscience Biotechnology Co., Ltd. (Wuhan, China). The anti-mouse IL-17A antibody (506904) was obtained from Biolegend Biotechnology Co., Ltd. (Shanghai, China).

### 2.3 UPLC‒Q‒TOF analysis

Characteristic components of HLPWS were characterized via ultrahigh-performance liquid chromatography (UPLC) coupled with quadrupole-flight mass spectrometry (Q-TOF-MS/MS, Agilent 1290 L, Agilent 6565 MS, Agilent Technologies, Santa Clara, CA, United States). The chromatographic column used was a Zorbax eclipse Plus C18 column with a narrow bore RR (2.1 × 100 mm, 1.8 µm) maintained at 45°C. The samples were diluted 50 times in 20% aqueous acetonitrile and centrifuged at 14,000 rpm for 10 min, after which the supernatants were injected. The mobile phase was composed of 0.1% formic acid in water (mobile phase A) and 0.1% formic acid in acetonitrile (mobile phase B) with a gradient elution program [5% B→95% B (0–60 min)] and 95% B [60–65 min)] at a flow rate of 0.5 mL/min and an injection volume of 0.5 μL. The system was operated in positive or negative scan mode. The instrument parameters were 3,500 V (−) or 3,500 V (+) capillary voltage, 11 L/min flow rate at 300°C, 350°C sheath gas temperature, 135 V cataclastic voltage, 45 psi nebulizer pressure, MS scan (MS mass range: 100–1700 m/z) and four spectra of the scan speed.

### 2.4 Animal experimental design

Thirty SPF-grade C57BL/6 mice (male, 8 weeks old, 20 g) were purchased from Guangdong Laboratory Animal Center, Guangdong, China (No. 44007200102395). All the mice were housed under a 12 h light/dark cycle and had free access to food and water. The animal study was approved by the Animal Ethics and Welfare Committee (AEWC) of Zhongshan Hospital of Traditional Chinese Medicine (AEWC-2023002), and the experimental procedures were carried out in strict accordance with the principles of Laboratory Animal Care and the guidelines. The mice were randomly divided into five groups: the control group, 2.25% DSS group, 2.25% DSS + SASP group (200 mg/kg), 2.25% DSS + HLPWS low-dose group (HLPWS-L, 10 mg/kg) and 2.25% DSS + HLPWS high-dose group (HLPWS-H, 20 mg/kg). The administered low dose of HLPW was calculated according to the recommended human dose [71 g/70 kg/day × conversion factor (9.1)]. Mice in the SASP and HLPWS groups were treated daily by oral gavage from days 1–14. The mice were induced with 2.25% DSS (W/V) for 7 days (from day 8 to day 14, free access to water). After the last treatment, all the mice were anaesthetized with isoflurane. Blood samples were obtained from the orbital sinus, and serum was collected by centrifugation for 10 min at 4,000 rpm and 4°C and stored at −80°C until use. The colons were collected and photographed. Parts of the colon were fixed in 4% paraformaldehyde (PFA) for further analysis, and the others were stored at −80°C.

### 2.5 Assessment of disease activity index (DAI)

The DAI for colitis was determined as previously described and was based on body weight changes, rectal bleeding, and stool consistency (Zhan et al., 2022). The detailed scoring system for DAI is shown in Table 1.

**TABLE 1:** Scoring system for DAI.

Weight loss (%)	Stool consistency	Blood stool	Score
0–1	Normal	None	0
1–5	Soft stool	Slight occult blood	1
5–10	Paste stool	Occult blood	2
10–15	Loose stool	Bleeding	3
≥15	Diarrhea	Gross bleeding	4

### 2.6 Enzyme-linked immunosorbent assay (ELISA) and biochemical analyses

The concentrations of MDA, SOD, CAT and GSH in the colons were determined via biochemical analysis kits according to the manufacturer’s instructions. Colon tissues (50 mg of each sample) were homogenized with cold phosphate-buffered saline (PBS, 0.01 M, pH 7.4, 1:10 w/v). The samples were subsequently centrifuged at 5,000 × g for 10 min at 4°C, after which the supernatants were collected for biochemical analysis. The levels of IL-6, IL-1β, LPS, IL-18, ROS, MPO, and DAO in colon tissues and of IL-22, IL-23, and IL-17 in serum were evaluated via ELISA following the manufacturer’s instructions.

### 2.7 Western blot analysis

Protein samples from colon tissues were extracted with RIPA lysis buffer supplemented with 1% protease inhibitor and phosphatase inhibitor (Dai et al., 2023). Protein concentrations were measured with a bicinchoninic acid (BCA) protein assay kit (#23225, Thermo, United States). Fifty micrograms of protein were separated via sodium dodecyl sulfate‒polyacrylamide gels and transferred onto polyvinylidene difluoride (PVDF) membranes (Merck Millipore Ltd., IPVH00010, Darmstadt, Germany). The transferred membranes were blocked with 5% skim milk in PBS containing 0.1% Tween-20 (PBST) (P0216, Beyotime Biotechnology, Shanghai, China), washed with PBST (0.1% Tween-20 in PBS) and incubated with primary antibodies at 4°C overnight. After being washed with PBST 3 times, the membranes were incubated with secondary antibodies conjugated with horseradish peroxidase (HRP) (1:10,000) at room temperature. The protein bands were visualized via enhanced chemiluminescence (ECL) reagent (Thermo Fisher Scientific) with a chemiluminescence imaging system (Bio-Rad, CA, United States). Quantification of the protein bands was performed with ImageJ software.

### 2.8 Hematoxylin and eosin (HE) staining

HE staining of colon tissues was performed according to previous methods with minor modifications (Yeganeh et al., 2018). Colon tissues were fixed with 4% PFA for 24 h, dehydrated with gradient ethanol, and paraffin embedded. Then, the tissues were slid into sections (∼4 μm thick) and stained with H&E. The morphological changes in the tissues were captured via an optical microscope (Nikon Corporation, ECLIPSE Ti2-A, Tokyo, Japan; magnification, ×200 for colon tissues).

### 2.9 Immunohistochemistry (IHC)

IHC analysis was performed to examine the protein expression of MUC2, cleaved caspase-3 and p-H2AX in colon tissues (Kim et al., 2022). Briefly, 4 μm sections of paraffin-embedded samples were treated with 3% H_2_O_2_ at room temperature to deactivate the enzyme. The samples were subsequently heated in 10 mM sodium citrate buffer (pH 6.0) for 10 min, followed by cooling to room temperature. The sections were blocked with normal goat serum and then incubated with anti-MUC2, anti-p-H2AX and anti-cleaved caspase-3 antibodies (1:200) at 4°C overnight, followed by incubation with biotinylated secondary antibodies. The expression levels of MUC2, p-H2AX and cleaved-caspase three in colon tissues were captured via optical microscopy (×200 magnification for colon tissues).

### 2.10 Flow cytometry

The percentages of Treg and Th17 cells were measured via flow cytometry. To analyse the percentage of Tregs, the splenic single-cell suspension was incubated with the FITC-conjugated anti-mouse CD4 antibody and anti-mouse CD25 antibody complex at 4°C for 45 min. After cell immobilization and permeabilization with the fixation/permeabilization concentrate, the cells were incubated with the PE-conjugated anti-mouse Foxp3 antibody in the dark for 60 min. For Th17 cell percentage detection, the splenic single-cell suspension was stimulated with 1 μL of PMA/ionomycin mixture and 1 μL of BFA/monensin mixture (250×) at 37°C for 4 h. Then, the samples were incubated with the anti-mouse CD4 antibody at 4°C for 15 min. After fixation and permeabilization, the samples were incubated with the PE-conjugated anti-mouse IL-17A antibody in the dark for 15 min. Finally, all the samples were assessed via flow cytometry, and the results were analysed via Cytoscape software.

### 2.11 Transferase-mediated dUTP nick-end labelling (TUNEL) assay

The TUNEL assay was performed by labelling the 3′-end of the fragmented DNA from apoptotic colon tissue. Briefly, the colon tissue was deparaffinized, rinsed with PBS, and analysed via a TUNEL apoptosis detection kit according to the manufacturer’s instructions (Beyotime, Shanghai, China). FITC-labelled TUNEL-positive cells were imaged via a fluorescence microscope (IX-71; Olympus, Tokyo, Japan).

### 2.12 Statistical analysis

SPSS (IBM SPSS, United States) was used to analyse the data statistically. All the data are expressed as the means ± standard errors of the means (SEMs). The significant differences between the two groups were compared by *Student’s t tests*. *p* < 0.05 was considered to indicate statistical significance.

## 3 Results

### 3.1 Identification of significant components of HLPWS extract via UPLC-Q-TOF

**FIGURE 1:**
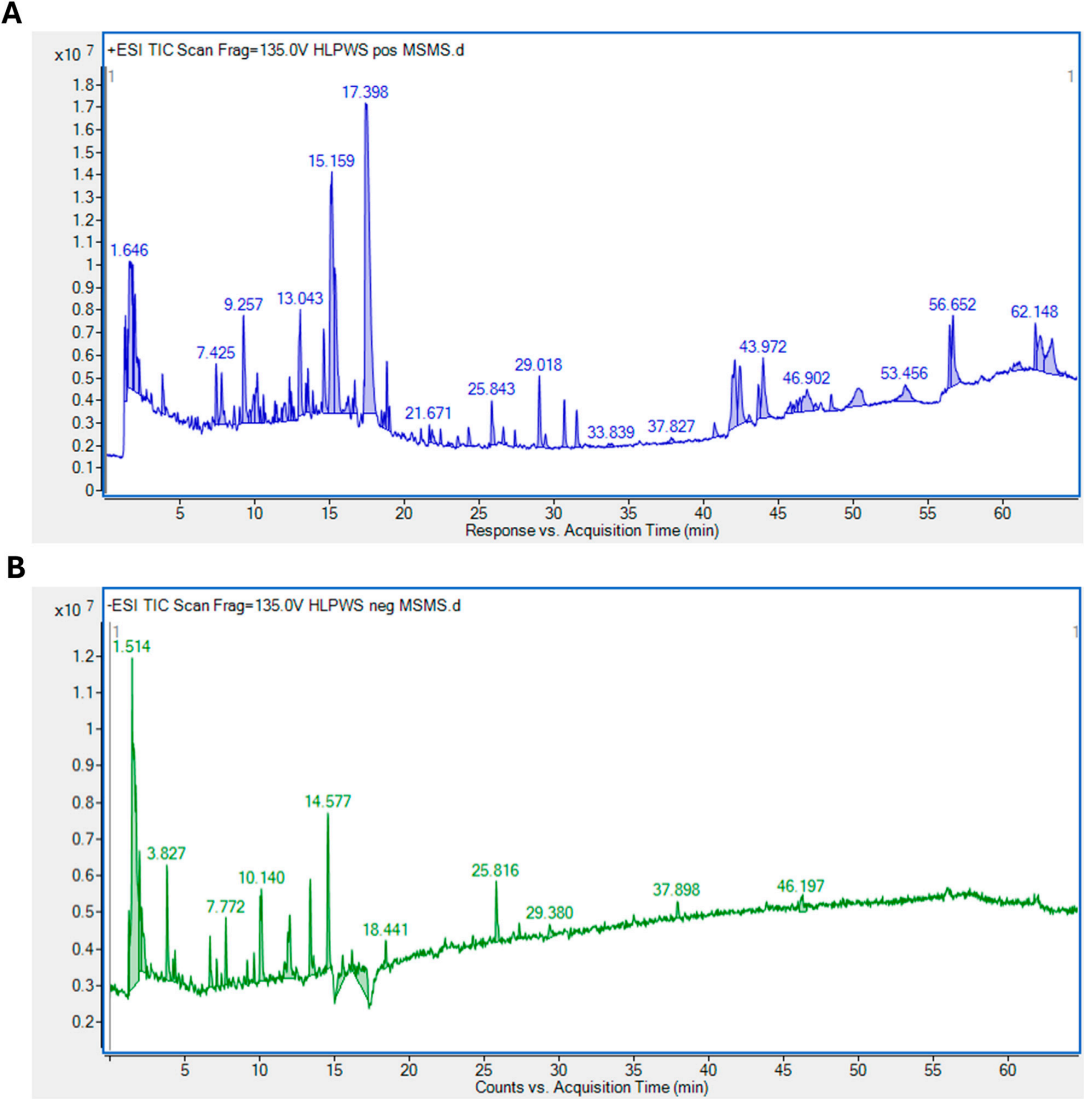
**(A)** Positive ions mode, **(B)** Negative ions mode.

As shown in Figure 1, a total of 93 major compounds of *HLPWS* were identified by UPLC-Q-TOF in positive mode. In addition, 48 major compounds were identified in negative mode. After removing duplicate components, 122 compounds, including amino acids, alkaloids, esters, phenolics, phenylpropanoids, coumaroids, flavones, organic acids, terpenoids, saccharides and others, were identified. Detailed information on all the compounds detected by UPLC‒Q‒TOF is shown in Supplementary Tables S1, S2.

### 3.2 HLPWS alleviated DSS-induced UC symptoms

**FIGURE 2:**
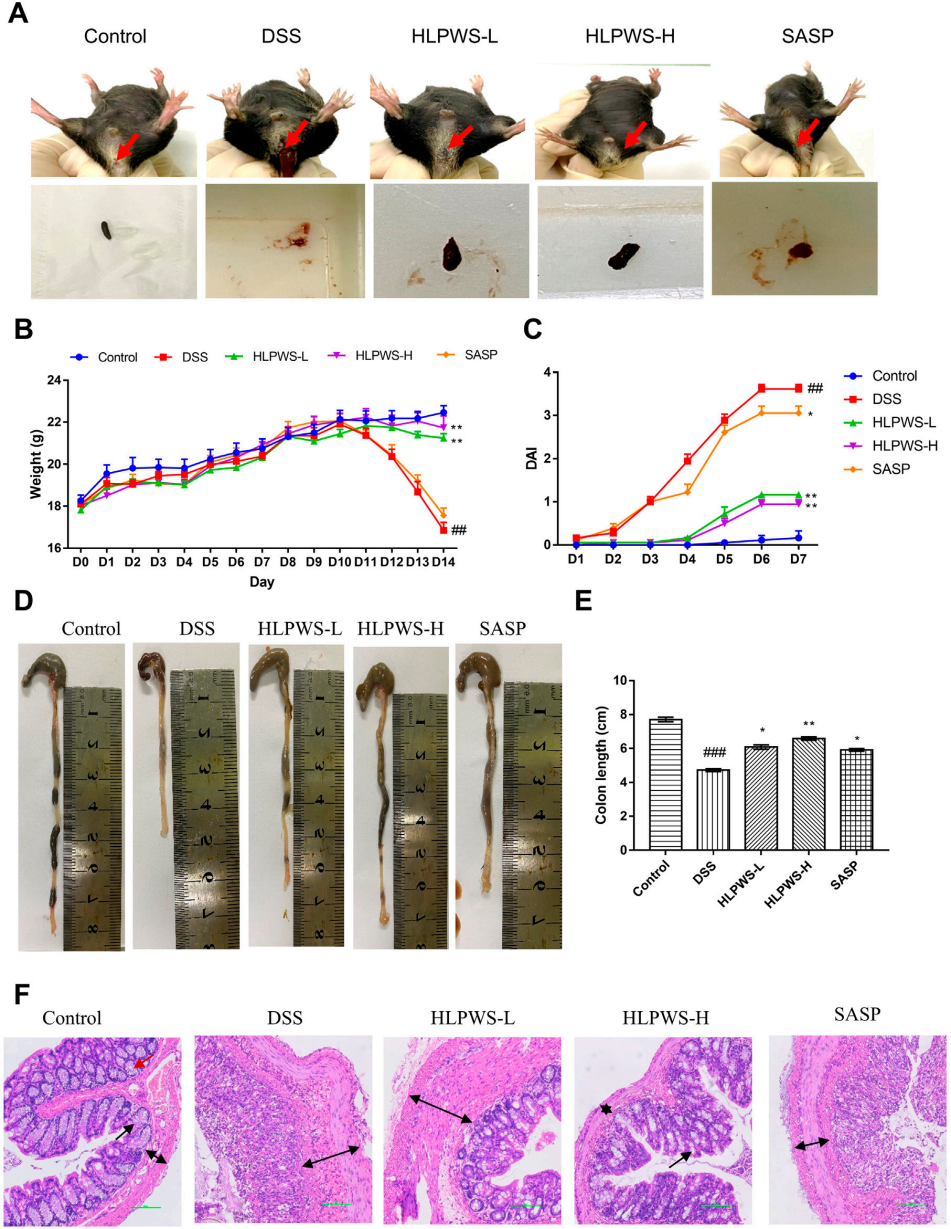
HLPWS alleviated DSS-induced UC symptoms. **(A)** Representative photographs of mouse feces. Effects of HLPWS on the body weight **(B)** and DAI **(C)** of colitis model mice. **(D)** Representative photographs of the colon. **(E)** Effects of HLPWSs on the colon length of UC mice. **(F)** Colon tissues were stained with HE for histopathological analysis (original magnifications, ×200; scale bar = 100 μm). The results are expressed as the means ± SEM. ^##^ *p* < 0.01, ^###^ *p* < 0.001 vs. Control group; ^***^ *p* < 0.001, ^**^ *p* < 0.01, ^*^ *p* < 0.05 vs. DSS group.

To evaluate the therapeutic efficacy of HLPWS, a 2.25% DSS-induced UC mouse model was utilized, with parameters including body weight, DAI score, and colonic length measured. Compared with those in the control group, the mice in the DSS model group presented severe UC symptoms, such as rectal bleeding, and diarrhea (Figure 2A), body weight loss (Figure 2B), all of which were significantly alleviated in the HLPWS- and SASP-treated groups. In addition, HLPWS and SASP effectively reversed the increased DAI scores (Figure 2C) and shortened the colonic length (Figures 2D, E) caused by DSS.

The effect of HLPWS on the changes in histopathological morphology of colon tissues from UC mice was further assessed via HE staining (Figure 2F). The colonic tissue of the mice in the control group displayed a well-defined structure and consistent distribution in the intestinal mucosal layer and adventitia. Following DSS induction, there was a significant alteration in histopathological morphology, characterized by a disrupted mucosal layer, absence of goblet cells, and expansion of the muscular and mucosal layers. Nevertheless, HLPWS therapy effectively attenuated the decrease in pathological alterations in colon tissues. The intestinal mucosa goblet cells were rejuvenated, and the columnar cells were organized in a systematic manner among the HLPWS-treated mice. Together, these observations suggest that HLPWS efficiently relieved UC symptoms in DSS-induced mice. Notably, the effects of HLPWS on body weight loss, DAI score, and histopathological morphology of colon tissues were superior to those of SASP.

### 3.3 HLPWS protected the intestinal barrier in UC mice

**FIGURE 3:**
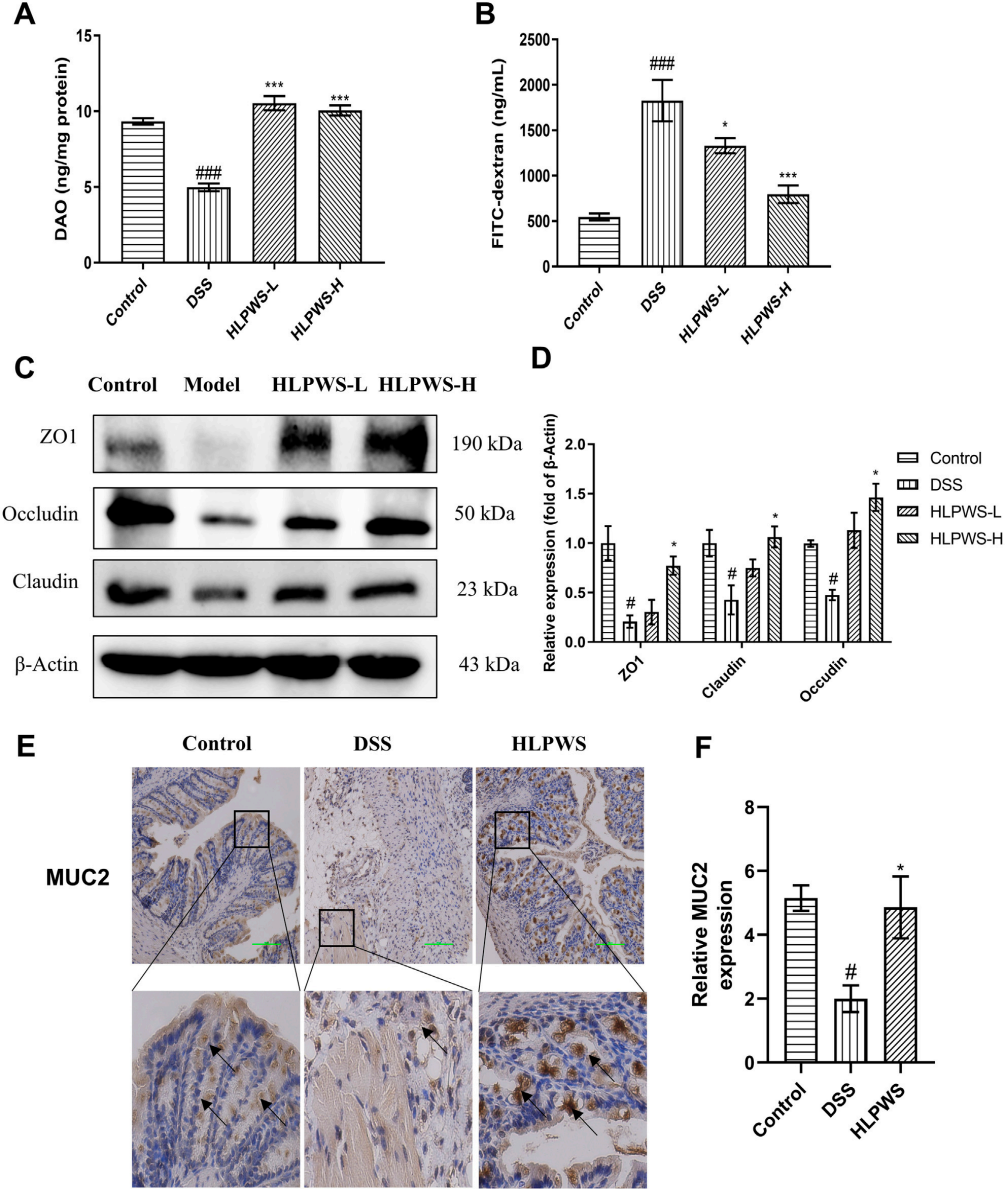
HLPWS Enhanced the Intestinal Barrier of UC Mice. **(A)** Effect on the DAO concentration of the colonic homogenate. **(B)** Evaluation of TJ permeability by FITC-dextran. **(C)** The expressions of the tight junction proteins ZO1, Claudin and occludin in colon tissues were evaluated by Western blotting. **(D)** Quantitative analysis of the Western blot results normalized to that of β-actin. **(E)** Expression of MUC2 in the colon tissues of UC mice was detected by IHC (original magnification, ×200; scale bar = 100 μm). **(F)** Quantitative analysis of the IHC results (*n* = 3). The results are expressed as the mean ± SEM. ^###^p < 0.001, ^##^p < 0.01, ^#^p < 0.05 vs. the control group; ***p < 0.001, *p < 0.05 vs. the DSS group.

The impact of HLPWS on the structure and function of the intestinal barrier in UC mice was assessed by analyzing the levels of DAO and FITC-dextran in the colon and the protein expressions of ZO-1, occludin, and claudin. Reduced DAO in the intestinal mucosa and elevated serum levels of FITC-dextran indicate damage to the intestinal barrier. The results revealed a significant decrease in DAO activity in the colon of DSS-treated mice compared with that in the control group, which was effectively reversed by HLPWS treatment (Figure 3A). Moreover, the levels of FITC-dextran were elevated in the model group but were dose-dependently reduced by HLPWS (Figure 3B). Western blot analysis revealed that HLPWS effectively upregulated the DSS-induced reduction in the protein expressions of ZO-1, occludin, and claudin in colon tissues, which was consistent with the H&E staining results (Figures 3C, D). Additionally, MUC2, a crucial protein in the mucin family secreted by goblet cells in the intestinal tract, was decreased in UC model mice, as detected by immunohistochemistry (Figures 3E, F). HLPWS reversed the decrease in MUC2 levels in UC model mice induced by DSS, suggesting its positive impact on the permeability of the intestinal barrier.

### 3.4 HLPWS inhibited DSS-induced colon cell apoptosis

**FIGURE 4:**
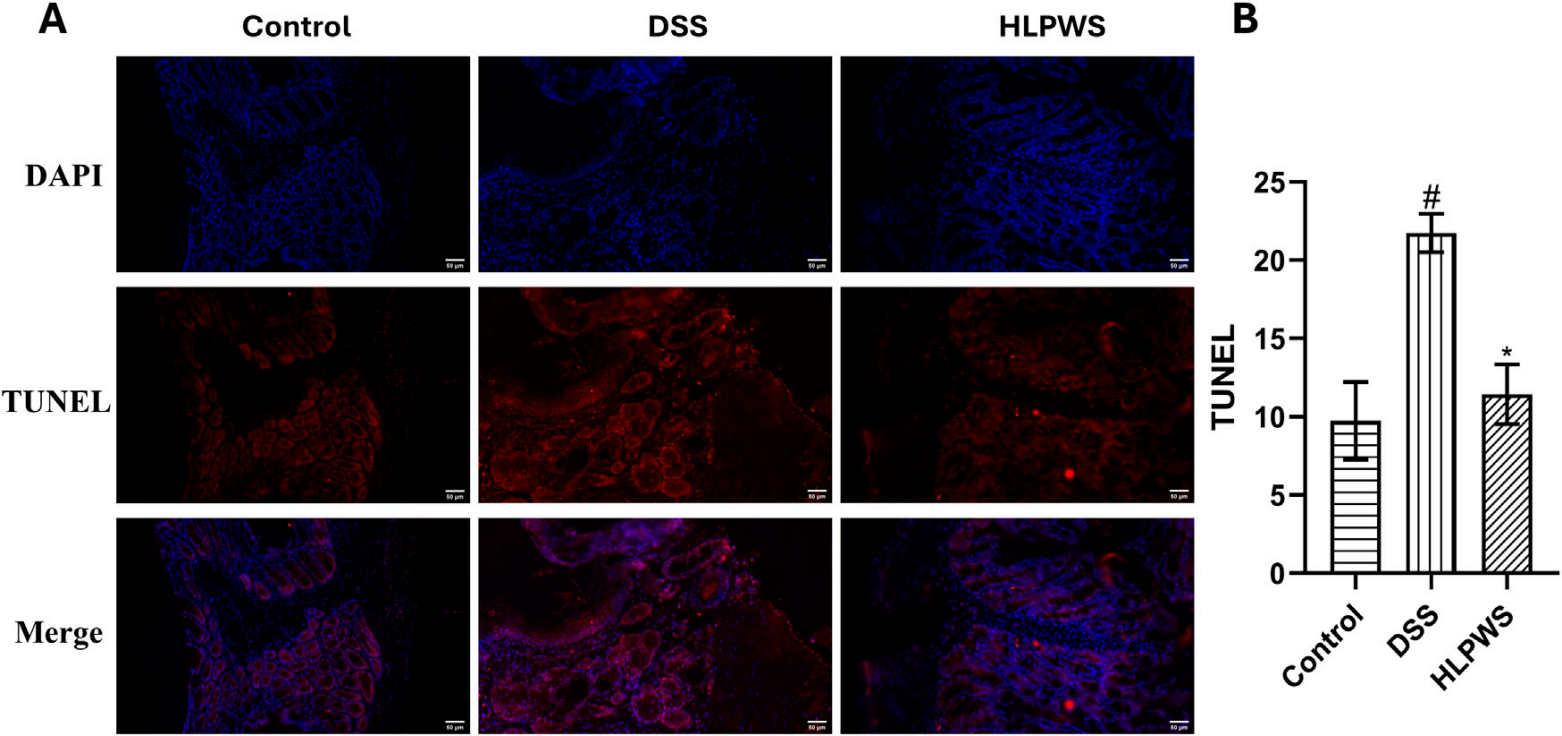
Effect of FLPWS on colon apoptosis in UC mice. **(A)** Evaluation of colon apoptosis via the TUNEL assay. (original magnifications, ×400, scale bar = 50 μm). **(B)** Quantitative analysis of the TUNEL results (*n* = 3). The results are expressed as the means ± SEM. #*p* < 0.05 vs. the control group; *p < 0.05 vs. the DSS group.

**FIGURE 5:**
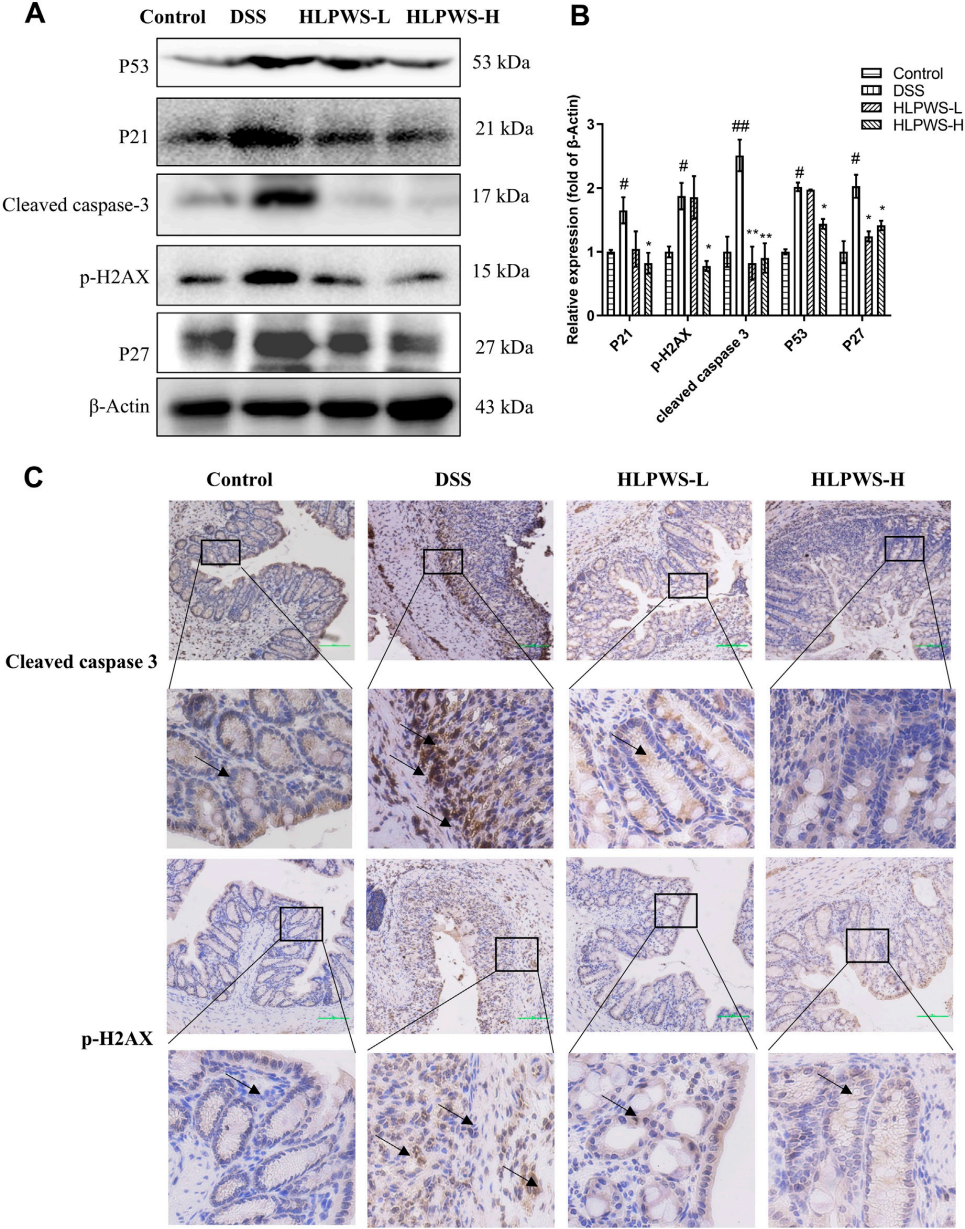
HLPWS inhibited apoptosis-related proteins. **(A)** Protein expression of P53, P21, cleaved caspase 3, p-H2AX, and P27 in colon tissues was determined by Western blotting. **(B)** Quantitative analysis of the Western blot results (*n* = 3). **(C)** Expression of cleaved caspase three and p-H2AX in the colon tissues of UC mice detected by IHC (original magnifications, ×200; scale bar = 100 μm). The results are expressed as the means ± SEM. ^##^ *p* < 0.01, ^#^ *p* < 0.05 vs. the control group; ^**^ *p* < 0.01, ^*^ *p* < 0.05 vs. the DSS group.

To further investigate the protective effect of HLPWS on colon cells in DSS-induced UC mice, apoptotic cells and related protein expressions were assessed via TUNEL staining, Western blot, and IHC. The results revealed a significant increase in the number of apoptotic cells in the colon tissues of the DSS-treated group compared with the control group (Figures 4A, B). In the group treated with HLPWS, there was a dose-dependent decrease in the number of apoptotic cells. Moreover, the elevated expression levels of P53, P21, P27, cleaved caspase 3, and p-H2AX in the colon tissues of DSS-stimulated mice were significantly decreased by HLPWS treatment (Figures 5A, B). Additionally, an IHC assay revealed that HLPWS effectively suppressed the increased expression of cleaved caspase-3 and p-H2AX induced by DSS, which was consistent with the Western blot findings (Figure 5C). Taken together, these results suggest that HLPWS expressions successfully defend against DSS-induced apoptosis in colon cells.

### 3.5 HLPWS reduced abnormal oxidative stress in UC mice

**FIGURE 6:**
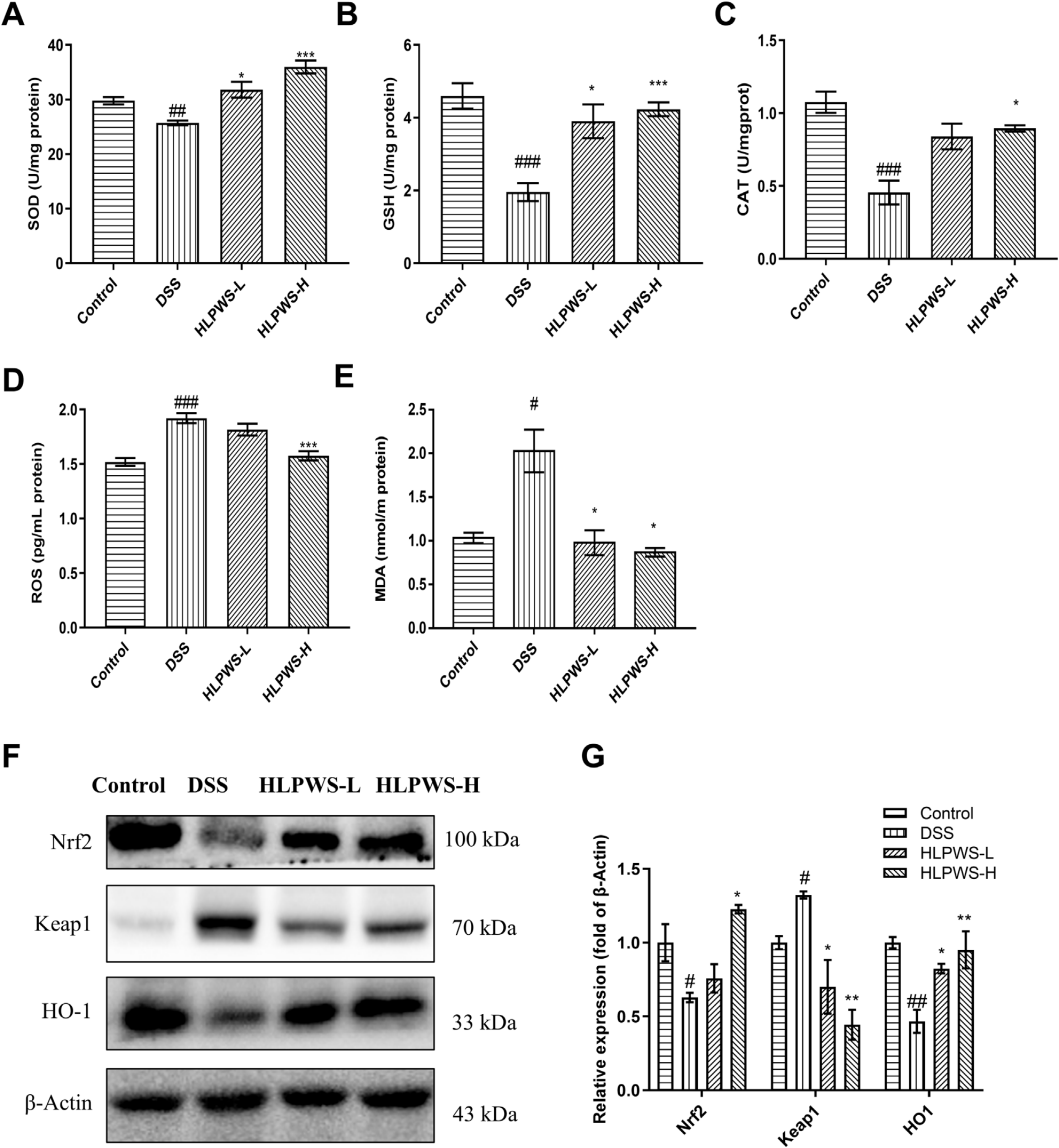
HLPWS reduced abnormal oxidative stress in UC mice. Effects of HLPWS on the levels of SOD **(A)**, GSH **(B)**, CAT **(C)**, ROS **(D)** and MDA **(E)** in colon homogenates, as detected via ELISA and biochemical kits. **(F)** Expression of Keap1, HO-1 and Nrf2 in the colon tissues of UC mice was evaluated by Western blotting. **(G)** Quantitative analysis of the Western blot results (*n* = 3). The results are expressed as the means ± SEM. ^###^ *p* < 0.001, ^##^ *p* < 0.01, ^#^ *p* < 0.05 vs. the control group; ***p < 0.001, **p < 0.01, *p < 0.05 vs. the DSS group.

Oxidative stress plays a critical role in the onset of UC. To evaluate the antioxidative effects of HLPWS in DSS-induced colitis, the levels of SOD, ROS, GSH, MDA, and CAT were detected by ELISA. The results revealed a significant decrease in the levels of SOD, GSH, and CAT (Figures 6A–C), important parameters for antioxidation, whereas the levels of ROS and MDA (Figures 6D, E) significantly increased after DSS induction (*p* < 0.01 and *p* < 0.05, respectively, compared with those in the control group). Following HLPWS treatment, the effects of DSS on SOD, ROS, GSH, MDA, and CAT were significantly reversed (*p* < 0.05 compared with those of the model group). Furthermore, this study evaluated the effects of HLPWS on the expression of oxidative stress-related proteins. These findings suggested that DSS increased Keap1 expression while reducing Nrf2 and HO-1 expression. Conversely, following HLPWS treatment, a significant reduction in Keap1 levels in colon tissues was observed, accompanied by a notable increase in Nrf2 and HO-1 levels (Figures 6F, G). These results indicate that HLPWS enhances antioxidative activity in the colons of UC model mice.

### 3.6 HLPWS inhibits inflammation in UC mice

**FIGURE 7:**
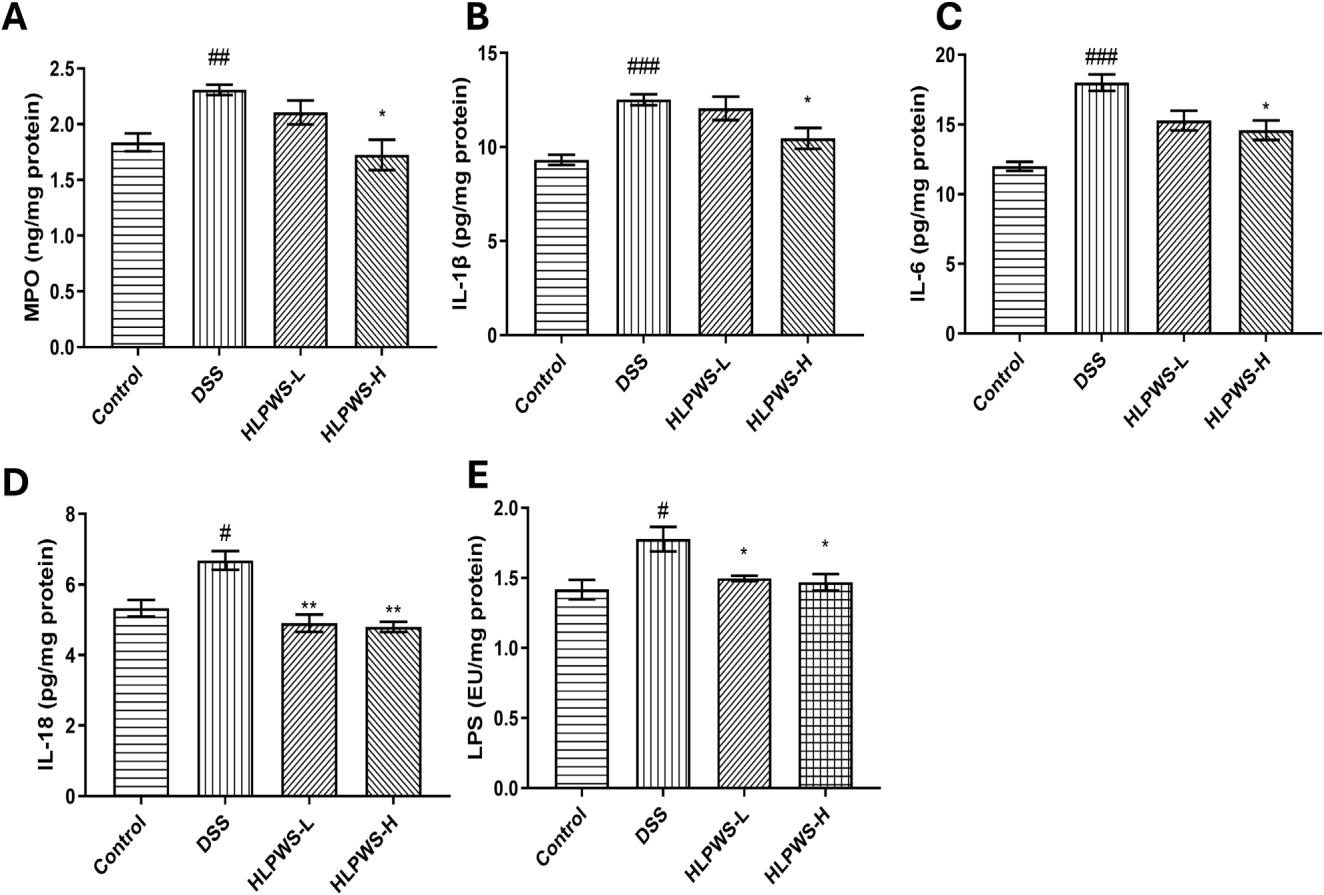
HLPWS inhibits inflammation in UC mice. Effects of HLPWS on the levels of MPO **(A)**, IL-1β **(B)**, IL-6 **(C)**, IL-18 **(D)**, and LPS **(E)** in colon homogenates. The results are expressed as the means ± SEM. ^###^ *p* < 0.001, ^##^ *p* < 0.01, ^#^ *p* < 0.05 vs. the control group; **p < 0.01, *p < 0.05 vs. the DSS group.

Myeloperoxidase (MPO) is a critical indicator of the progression of inflammation in the intestines. Figure 7A clearly shows that the levels of MPO in colon tissue significantly increased in DSS-induced UC mice. HLPWS effectively reduced MPO levels in a dose-dependent manner. Moreover, an ELISA was used to analyse the levels of the proinflammatory cytokines IL-1β, IL-6, IL-18, and LPS in colon tissues. Compared with those in the model group, the HLPWS group presented notable reductions in the levels of IL-1β, IL-6, IL-18, and LPS in colon tissue (Figures 7B–E).

**FIGURE 8:**
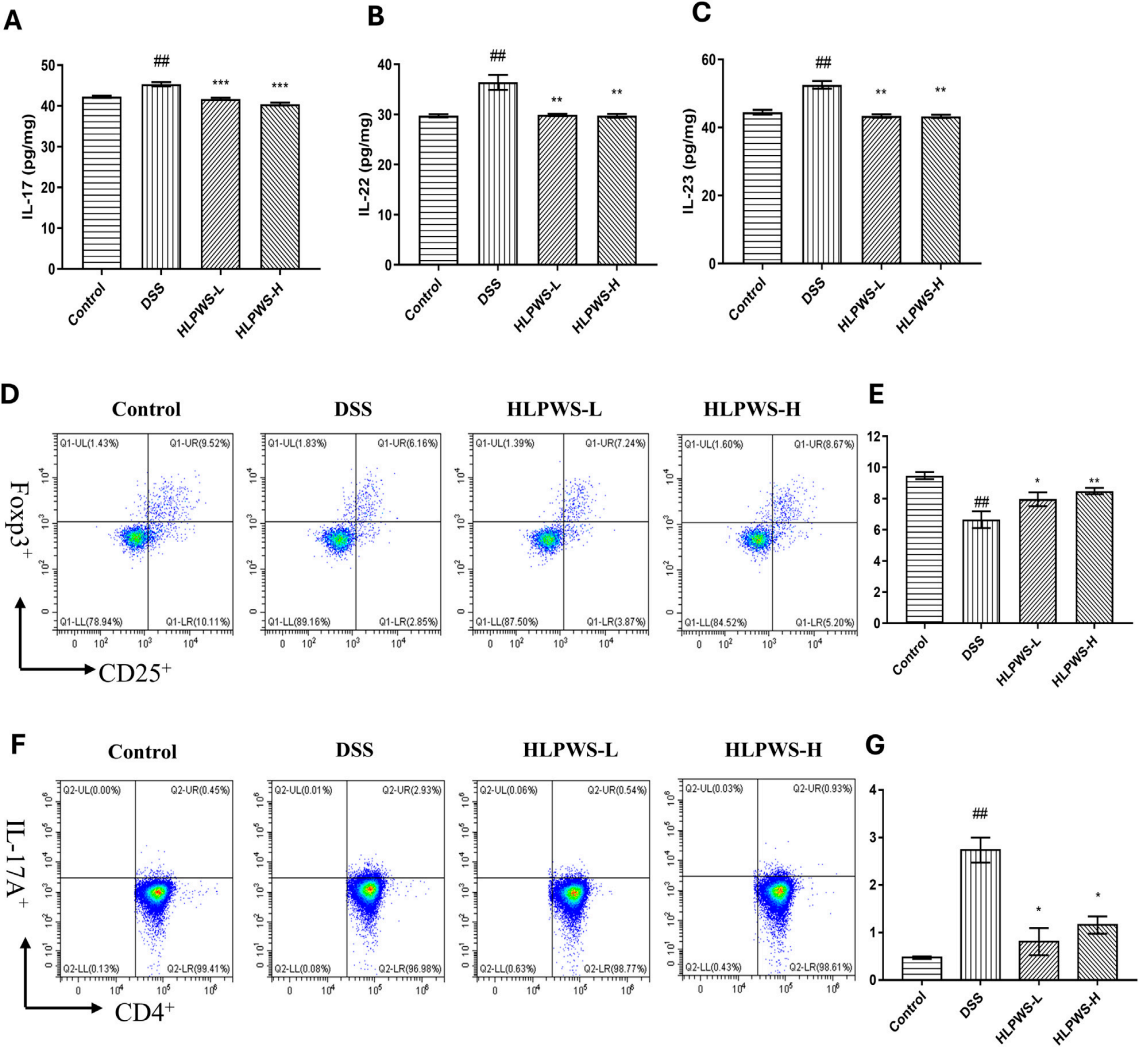
HLPWS restored the immune balance. Effects of HLPWS on the levels of IL-17 **(A)**, IL-22 **(B)**, and IL-23 **(C)** in colon homogenates, as detected via ELISA. Representative photographs of CD4^+^CD25^+^Foxp3^+^ (Treg) cells in the spleen analysed by flow cytometry **(D)** and quantitative analysis of the percentage of Treg cells **(E)**. Representative photographs of CD4+IL-17A (Th17) cells in the spleen analysed by flow cytometry **(F)** and quantitative analysis of the percentage of Th17 cells **(G)**. The results are expressed as the mean ± SEM (*n* = 3). ^##^ *p* < 0.01 vs. the control group; ***p < 0.001, **p < 0.01, *p < 0.05 vs. the DSS group.

The IL23/IL17 axis is known to play a crucial role in the development of UC by promoting Th17 cell activation and cytokine-mediated immune responses (Noviello et al., 2021). The results revealed that the levels of IL-17, IL22, and IL23 in the serum were elevated in the DSS group compared with those in the control group and decreased after HLPWS administration (Figures 8A–C). Th17 cells produce the proinflammatory cytokine IL-17A and are implicated in various autoimmune conditions, including UC. Conversely, Treg cells function as suppressors of immune responses by releasing anti-inflammatory cytokines (Lee, 2018). These cell types play opposing roles in inflammation and collaborate to maintain the intestinal immune balance. Flow cytometry analysis revealed a significant increase in Th17 cells in the spleens of DSS-induced UC mice compared with those in the control group. The proportion of Th17 cells decreased significantly in the HLPWS-H group, which was consistent with the ELISA results. In contrast, splenic Treg cells were significantly reduced in the DSS group but increased after HLPWS-H treatment, indicating that HLPWS has potent anti-inflammatory effects on DSS-induced UC mice by modulating proinflammatory cytokines and rebalancing Treg and Th17 cell populations.

## 4 Discussion

The primary therapeutic target for UC is the repair of intestinal epithelial barrier dysfunction resulting from inflammation and oxidative stress. Numerous studies have suggested that inflammation and oxidative stress can compromise the integrity of the IEB, thus exacerbating the progression of UC (Zheng et al., 2023). Current pharmacotherapies predominantly aim to prevent further colon damage; however, they are limited in their capacity to repair the damaged colon and often negatively impact quality of life due to side effects. According to traditional Chinese medicine, HLPWS is believed to clear heat and detoxify, dry dampness, and invigorate the spleen, making it widely utilized in the treatment of gastrointestinal diseases. The current research demonstrated that HLPWS had a significant positive effect on symptoms of UC by increasing both body weight and colon length while simultaneously decreasing the DAI score and improving pathological changes in colon tissues. Additionally, HLPWS notably improved intestinal barrier integrity and reduced the number of apoptotic cells in the colons of mice with UC. These beneficial effects of HLPWS were attributed to its ability to repair inflammatory damage and mitigate oxidative stress within the colon, thereby providing additional potential therapeutic options for UC management.

Abnormal immune responses in the intestinal system play a key role in the development of UC. The increased release of inflammatory cytokines, including IL-6 and LPS, initiates a persistent immune reaction, impacting the distribution of tight junction proteins and potentially damaging the intestinal epithelial cell structure (Hou et al., 2020). Moreover, IL-6 plays a crucial role in regulating the functions of T cells, such as differentiation, activation, and programmed cell death, thereby impacting the equilibrium of proinflammatory T-cell populations (such as Th17 cells) and immunosuppressive regulatory T cells (Li et al., 2018). IL-23, which is part of the IL-12 cytokine group, has the ability to increase the growth of harmful Th17 cells via different methods. Immune cells that are activated secrete inflammatory cytokines that then control various cell types, promoting the generation of IL-1β and IL-18 by mononuclear phagocytes and intestinal epithelial cells and worsening inflammation (Tao et al., 2018). The IL-17 and IL-23 pathways can stimulate the production of various inflammatory molecules, such as TNF, IFNγ, IL22, lymphotoxin, IL-1β, and LPS (Schmitt et al., 2021). Maintaining a balance between Th17 and Treg cells is critical, as they engage in reciprocal inhibition to regulate immune homeostasis. An imbalance in these cells can lead to autoimmune disorders such as UC (Yan et al., 2020). Our research revealed that HLPWS inhibited the release of the inflammatory factors IL-17, IL-22, and IL-23, decreases Th17 cell differentiation, enhances Treg cell differentiation, and ultimately enhances the immune response in UC mice. Moreover, the restoration of the Th17/Treg balance may indicate that HLPWS has the potential to modulate the immune response beyond the acute phase of UC, which is significant given that UC is a chronic condition characterized by cycles of remission and relapse. By promoting immune tolerance through Treg regulation, HLPWS may facilitate a more sustained remission, ultimately reducing the frequency and severity of UC flares. However, further studies are necessary to fully elucidate the underlying mechanisms and to confirm the long-term effects of HLPWS on the prevention of UC relapse.

Inflammation and oxidative stress are mutually reinforcing, and inflammation increases ROS production, which in turn exacerbates inflammation. Oxidative stress is a critical pathogenic element in UC. External substances can easily disturb the redox balance in the colon, leading to oxidative stress. Growing evidence suggests that oxidative stress plays a role in promoting UC (Chen et al., 2021). Oxidative stress is characterized by an imbalance between the production of free radicals, mainly ROS, and their elimination by antioxidant defense systems present in tissues and body fluids (Tahvilian et al., 2020). This imbalance can disrupt the cystine/glutamate antiporter, reducing cystine intake and blocking GSH synthesis (Lewerenz et al., 2013). DSS can increase the production of oxygen free radicals such as MDA and ROS, decreasing the levels of GSH and SOD and resulting in oxidative stress in the colon. Excessive MDA can chemically modify proteins, nucleic acids, and lipids, leading to cell death or structural damage (Del Rio et al., 2005). The Keap1‒Nrf2 signalling pathway plays a crucial role in protecting cells from both endogenous and exogenous oxidative stresses (Yamamoto et al., 2018). Nrf2, a transcription factor involved in detoxification and antioxidant processes, regulates GSH metabolism. Keap1, a redox-sensitive member of the BTB-Kelch family, is responsible for degrading Nrf2 when oxidative stress occurs. When Nrf2 evades Keap1-induced degradation, it translocates to the nucleus and triggers a variety of genes reliant on ARE, including antioxidant and cytoprotective genes such as HO-1 and SOD. Our findings demonstrated that HLPWS significantly increased the levels of SOD and GSH, enhanced the expression of Nrf2 and HO-1, inhibited the expression of Keap1, and reduced the levels of ROS and MDA. These findings indicate that HLPWS may alleviate UC by suppressing oxidative stress.

Oxidative stress caused by overproduction of ROS is an important mechanism of endothelial cell death (Zheng et al., 2022). Oxidative stress has been shown to increase P53 transcriptional activity (Peuget et al., 2014). P53, often referred to as the ‘genome protector’, plays crucial roles, including the induction of apoptosis and cellular senescence (Lane, 1992). P53 directly regulates the transcription of P21, and P21 activation subsequently triggers apoptosis. Increased expression of the P21 variant amplifies P53 activity, thereby enhancing its proapoptotic effects (Engeland, 2022). Caspase-3, which is usually present in an inactive state within cells, becomes activated by P53 and P21, acting as a critical indicator of irreversible apoptosis (Chan et al., 2010). P53 can activate downstream target genes such as p21 and caspase-3, thereby triggering apoptosis (Cubillos-Rojas et al., 2017). Studies have also indicated that H2AX can activate P53, leading to cell cycle arrest and apoptosis. Our findings demonstrate that HLPWS significantly inhibits the protein expression of P53 and P21, as well as the expression of cleaved caspase three and p-H2AX, suggesting that HLPWS has the potential to inhibit cell damage and apoptosis induced by oxidative stress. Furthermore, p-H2AX is considered a well-known marker of DNA damage. The increased expression induced by HLPWS may predict long-term protective effects against the development of UC and could contribute to a reduction in relapse rates among patients. However, the impact of HLPWS on DNA repair mechanisms still requires further investigation.

The death and deterioration of intestinal epithelial cells play a direct role in dysfunction of the intestinal barrier, worsening UC conditions. A healthy intestinal epithelial barrier is maintained by tight junctions and adhesive linkages (Gustafsson and Johansson, 2022). Additionally, these cells produce a protective mucus layer called MUC2, which helps safeguard the intestinal barrier and preserves the balance of the microbiome (Liu et al., 2020). Our findings demonstrated that DSS led to a significant reduction in the protein levels of ZO1, Claudin-1, Occludin, and MUC2 in UC mice, indicating damage to and apoptosis of intestinal epithelial cells. Conversely, the administration of HLPWS effectively restored the protein levels of ZO1, Claudin-1, Occludin, and MUC2 in the colon, highlighting the reparative effects of HLPWS on the intestinal barrier.

While the study demonstrates the protective effect of HLPWS through amelioration of inflammatory response and oxidative stress, the underlying molecular mechanisms remain incompletely understood. Further research is required to elucidate the precise pathways through which HLPS exerts its protective effects. Furthermore, identifying the active components of HLPWS and their potential molecular targets may help to identify key therapeutic agents in future studies. And these mechanisms of action will be further verified in combination with samples in clinical therapeutic.

## 5 Conclusion

Our study revealed that HLPWS had the potential to inhibit intestinal epithelial cell apoptosis and alleviate colonic intestinal barrier damage by reducing oxidative stress and restoring the immune imbalance in DSS-induced UC mice. These findings provide novel insights into the therapeutic benefits of HLPWS within a UC mouse model and highlight its prospective application as a candidate drug for UC in clinical settings. HLPWS could serve as an adjunct therapy for UC patients, particularly in cases where conventional treatments prove insufficient or result in significant side effects.

## Data Availability

The original contributions presented in the study are included in the article/Supplementary Material, further inquiries can be directed to the corresponding author.

## Abbreviations

DAI, Disease activity index; DAO, Diamine oxidase; DSS, Dextran sulfate sodium; ECL, chemiluminescence; Elisa, enzyme-linked immunosorbent assay; HE, Hematoxylin and eosin; HLPWS, Huangliang pingwei San; HO-1, Heme oxygenase 1; IEB, Intestinal epithelial barrier; IHC, immunohistochemistry; Keap1, Kelch-like ECH-associated protein 1; MPO, myeloperoxidase; Nrf2, Nuclear factor E2-related factor 2; ROS, Reactive oxygen species; SOD, Superoxide dismutase; TCM, traditional Chinese medicine; TJ, Tight junction; UC, ulcerative colitis; ZO1, Zona Occludens 1.
