# Supplementary material for: Protective effect of Huanglian Pingwei San on DSS-induced ulcerative colitis in mice through amelioration of the inflammatory response and oxidative stress
Source: Front Pharmacol. 2024 Dec 4;15:1484532. doi: 10.3389/fphar.2024.1484532 (PMC11652202; doi:10.3389/fphar.2024.1484532)
Supplement: Supplementary file 1 [file Table1.DOC]

SUPPLEMENTARY TABLE 1. Identified compounds of HLWDT by UPLC/MS QTOF in the positiveion mode.

|  | **Name** | **Formula** | **Mass** | **RT** | **Height** | **CAS** |
| --- | --- | --- | --- | --- | --- | --- |
| 1 | L-Lysine | C6 H14 N2 O2 | 146.1056 | 1.377 | 3786 | 56-87-1 |
| 2 | L(+)-Arginine | C6 H14 N4 O2 | 174.1125 | 1.423 | 346783 | 74-79-3 |
| 3 | L-Aspartic Acid | C4 H7 N O4 | 133.0375 | 1.49 | 5872 | 56-84-8 |
| 4 | Trigonelline | C7 H7 N O2 | 137.048 | 1.551 | 76592 | 6138-41-6 |
| 5 | Adenine | C5 H5 N5 | 135.0544 | 1.673 | 26880 | 73-24-5 |
| 6 | Pipecolinic acid | C6 H11 N O2 | 129.079 | 1.698 | 54510 | 535-75-1 |
| 7 | L-Phenylalanine | C9 H11 N O2 | 165.079 | 1.824 | 74118 | 63-91-2 |
| 8 | L-Tyrosine | C9 H11 N O3 | 181.0741 | 1.925 | 20271 | 60-18-4 |
| 9 | Synephrine | C9 H13 N O2 | 167.095 | 1.976 | 718522 | 94-07-5 |
| 10 | L-Pyroglutamic acid | C5 H7 N O3 | 129.0427 | 2.218 | 14853 | 98-79-3 |
| 11 | Adenosine | C10 H13 N5 O4 | 267.097 | 2.252 | 84912 | 58-61-7 |
| 12 | Hydroxytyrosol | C5 H5 N5 O | 151.0496 | 2.366 | 39246 | 10597-60-1 |
| 13 | Guanosine | C10 H13 N5 O5 | 283.0919 | 2.395 | 117378 | 118-00-3 |
| 14 | 4-Hydroxycinnamamide | C9 H9 N O2 | 163.0633 | 2.543 | 45691 | 194940-15-3 |
| 15 | L-Leucine | C6 H13 N O2 | 131.0947 | 2.659 | 10761 | 61-90-5 |
| 16 | Hordenine | C10 H15 N O | 165.1153 | 2.996 | 18752 | 539-15-1 |
| 17 | Hypaphorine | C14 H18 N2 O2 | 246.1368 | 3.675 | 13602 | 487-58-1 |
| 18 | 5-Hydroxymethyl-2-Furaldehyde | C6 H6 O3 | 126.0319 | 3.803 | 7515 | 67-47-0 |
| 19 | 3β,7β,15β-trihydroxy-11-oxo-lanosta-8-en-24→20 lactone | C27 H40 O6 | 460.2807 | 4.612 | 70871 | 1694587-15-9 |
| 20 | Scopolin | C16 H18 O9 | 354.0946 | 5.397 | 55040 | 531-44-2 |
| 21 | 7-Hydroxycoumarin | C9 H6 O3 | 162.0316 | 5.405 | 7398 | 93-35-6 |
| 22 | L-Tryptophan | C11 H12 N2 O2 | 204.0901 | 5.832 | 126994 | 73-22-3 |
| 23 | N-trans-Feruloyltyramine | C18 H19 N O4 | 313.1315 | 7.257 | 89983 | 66648-43-9 |
| 24 | Methyl 4-Methoxycinnamate | C11 H12 O3 | 192.079 | 7.31 | 13777 | 3901-07-3 |
| 25 | Cryptochlorogenic acid | C16 H18 O9 | 354.095 | 7.522 | 42077 | 905-99-7 |
| 26 | 3-O-Caffeoylquinic acid methyl ester | C17 H20 O9 | 368.1102 | 7.798 | 19146 | 123483-19-2 |
| 27 | 7-Methoxycoumarin | C10 H8 O3 | 176.0474 | 7.812 | 41415 | 531-59-9 |
| 28 | Dauricine | C38 H44 N2 O6 | 624.3199 | 7.96 | 38703 | 524-17-4 |
| 29 | Tetrandrine | C38 H42 N2 O6 | 622.3051 | 8.296 | 90253 | 518-34-3 |
| 30 | Piperine | C17 H19 N O3 | 285.1358 | 8.328 | 33947 | 94-62-2 |
| 31 | 3-(4-Hydroxy-3-methoxyphenyl)propionic acid | C10 H12 O4 | 196.0732 | 8.499 | 12049 | 1135-23-5 |
| 32 | Aempferol-3-O-rutinoside | C27 H30 O15 | 594.1583 | 8.987 | 131371 | 17650-84-9 |
| 33 | Narcissoside | C28 H32 O16 | 624.1687 | 9.548 | 47086 | 604-80-8 |
| 34 | Naringin | C27 H32 O14 | 580.1784 | 9.69 | 17820 | 10236-47-2 |
| 35 | Troxerutin | C33 H42 O19 | 742.2294 | 10.146 | 111393 | 7085-55-4 |
| 36 | Tetrahydrojateorrhizine | C20 H23 N O4 | 341.1627 | 10.305 | 113172 | 13063-54-2 |
| 37 | Chrysophanol-1-O-b-D-glucoside | C21 H20 O9 | 416.1109 | 11.115 | 25690 | 4839-60-5 |
| 38 | Isofraxidin | C11 H10 O5 | 222.0529 | 11.319 | 22671 | 486-21-5 |
| 39 | Secoisolariciresinol β-D-glucoside | C26 H36 O11 | 524.2254 | 11.339 | 52793 | 63320-67-2 |
| 40 | Tetrahydroberberine | C20 H21 N O4 | 339.1472 | 11.496 | 356534 | 5096-57-1 |
| 41 | Chelidonine | C20 H19 N O5 | 353.1259 | 11.612 | 63718 | 476-32-4 |
| 42 | Neoeriocitrin | C27 H32 O15 | 596.1744 | 11.705 | 24870 | 13241-32-2 |
| 43 | 2''-O-Rhamnosylvitexin | C27 H30 O14 | 578.1638 | 11.714 | 48616 | 64820-99-1 |
| 44 | D-Tetrahydropalmatine | C21 H25 N O4 | 355.1784 | 11.846 | 339531 | 3520-14-7 |
| 45 | Liguirtigenin-7-O-D-apiosyl-4'-O-D-Glucoside | C26 H30 O13 | 550.1681 | 11.929 | 39435 | 19979-12-8 |
| 46 | Dihydrodaidzein | C15 H12 O4 | 256.0736 | 12.008 | 60388 | 17238-05-0 |
| 47 | Liquiritin | C21 H22 O9 | 418.1264 | 12.086 | 161625 | 551-15-5 |
| 48 | Scopoletin | C10 H8 O4 | 192.0423 | 12.1 | 19025 | 92-61-5 |
| 49 | Stylopine | C19 H17 N O4 | 323.1157 | 12.529 | 233131 | 84-39-9 |
| 50 | Groenlandicine | C19 H16 N O4 | 322.1081 | 12.636 | 230166 | 38691-95-1 |
| 51 | Tetrahydropalmatine | C21 H25 N O4 | 355.1786 | 13.313 | 39903 | 2934-97-6 |
| 52 | FMOC-L-Isoleucine | C21 H23 N O4 | 353.1628 | 13.891 | 283314 | 71989-23-6 |
| 53 | Jatrorrhizine | C20 H19 N O4 | 337.1316 | 14.172 | 137448 | 3621-38-3 |
| 54 | Episyringaresinol 4'-O-β-D-glncopyranoside | C28 H36 O13 | 580.2157 | 14.228 | 53997 | 137038-13-2 |
| 55 | Hispidulin | C16 H12 O6 | 300.0633 | 14.462 | 99748 | 1447-88-7 |
| 56 | Hesperetin | C16 H14 O6 | 302.0791 | 14.605 | 53354 | 520-33-2 |
| 57 | Neohesperidin | C28 H34 O15 | 610.1899 | 14.637 | 65174 | 13241-33-3 |
| 58 | Licoagroside D | C22 H24 O10 | 448.137 | 14.657 | 84342 |  |
| 59 | Hesperidin | C28 H34 O15 | 610.1907 | 14.672 | 749126 | 520-26-3 |
| 60 | Berberrubine | C19 H17 Cl N O4 | 321.1 | 15.023 | 185368 | 15401-69-1 |
| 61 | Coptisine | C19 H15 Cl N O4 | 320.095 | 15.154 | 5646721 | 6020-18-4 |
| 62 | Epiberberine | C20 H18 N O4 | 336.1257 | 15.205 | 3653233 | 6873-09-2 |
| 63 | (+)- Corydaline | C22 H27 N O4 | 369.1941 | 15.532 | 113426 | 518-69-4 |
| 64 | Isoliquiritin apioside | C26 H30 O13 | 550.1684 | 15.581 | 43080 | 120926-46-7 |
| 65 | Corynoline | C21 H21 N O5 | 367.142 | 16.374 | 329775 | 18797-79-0 |
| 66 | Palmatine | C21 H21 N O4 | 351.147 | 16.489 | 159537 | 3486-67-7 |
| 67 | Formononetin glucoside | C22 H22 O9 | 430.1265 | 16.599 | 253331 | 486-62-4 |
| 68 | Chrysin | C15 H10 O4 | 254.058 | 16.672 | 23772 | 480-40-0 |
| 69 | Linderane | C15 H16 O4 | 260.105 | 16.997 | 13689 | 13476-25-0 |
| 70 | 5,7,4’-Trihydroxy-8-methylflavanone | C16 H14 O5 | 286.0841 | 17.323 | 22865 | 916917-28-7 |
| 71 | Berberine | C20 H17 N O4 | 335.1198 | 17.443 | 15241908 | 2086-83-1 |
| 72 | Curcumol | C15 H24 O2 | 236.1777 | 20.334 | 5530 | 4871-97-0 |
| 73 | Ligustroflavone | C33 H40 O18 | 724.2211 | 21.692 | 68697 | 260413-62-5 |
| 74 | Germacrone | C15 H22 O | 218.1669 | 22.038 | 19350 | 6902-91-6 |
| 75 | Ganoderic acid DM | C30 H44 O4 | 468.3235 | 22.43 | 29586 | 173075-45-1 |
| 76 | Bicuculline | C20 H17 N O6 | 367.105 | 22.716 | 31484 | 485-49-4 |
| 77 | Curcumenol | C15 H22 O2 | 234.1619 | 23.408 | 8944 | 19431-84-6 |
| 78 | Icaritin | C20 H20 O7 | 372.121 | 24.392 | 220758 | 5240-95-9 |
| 79 | Tectochrysin | C16 H12 O4 | 268.0735 | 24.766 | 29873 | 520-28-5 |
| 80 | Glycyrrhetic acid 3-O-mono-b-D-glucuronide | C36 H54 O10 | 646.3711 | 25.849 | 26229 | 34096-83-8 |
| 81 | Glycyrrhizic acid | C42 H62 O16 | 822.4037 | 25.919 | 118761 | 1405-86-3 |
| 82 | 5,7,8,4'-Tetramethoxyflavone | C19 H18 O6 | 342.1105 | 26.684 | 205787 | 6601-66-7 |
| 83 | Methyl rosmarinate | C19 H18 O8 | 374.1008 | 29.248 | 14913 | 99353-00-1 |
| 84 | Glycycoumarin | C21 H20 O6 | 368.1262 | 30.102 | 11942 | 94805-82-0 |
| 85 | Bruceine | C20 H26 O9 | 410.1593 | 30.965 | 25558 | 21499-66-2 |
| 86 | Isosinensetin | C20 H20 O7 | 372.1207 | 31.506 | 10830 | 17290-70-9 |
| 87 | Smyrindioloside | C20 H24 O10 | 424.1355 | 31.668 | 8228 | 87592-77-6 |
| 88 | Sanggenon H | C20 H18 O6 | 354.1107 | 32.258 | 20664 | 86450-80-8 |
| 89 | Bavachinin | C21 H22 O4 | 338.1517 | 32.975 | 22510 | 19879-30-2 |
| 90 | 2-Chromanone | C9 H8 O2 | 148.0525 | 35.697 | 5465 | 119-84-6 |
| 91 | 5-O,8-Dimethylophiopogonanone B | C20 H22 O5 | 342.1471 | 41.87 | 118536 | 1234211-72-3 |
| 92 | Dibutyl phthalate | C16 H22 O4 | 278.1521 | 43.673 | 247521 | 84-74-2 |
| 93 | Camptothecin | C20 H16 N2 O4 | 348.1102 | 56.179 | 8237 | 7689/3/4 |

SUPPLEMENTARY TABLE 2. Identified compounds of HLWDT by UPLC/MS QTOF in the negativeion mode.

|  | **Name** | **Formula** | **Mass** | **RT** | **Height** | **CAS** |
| --- | --- | --- | --- | --- | --- | --- |
| 1 | L(+)-Arginine | C6 H14 N4 O2 | 174.1118 | 1.398 | 53781 | 74-79-3 |
| 2 | D-Glucosamine | C6 H13 N O5 | 179.0794 | 1.425 | 17838 | 28905-10-4 |
| 3 | D-(+)-Lactose | C6 H12 O6 | 180.0634 | 1.468 | 21786 | 63-42-3 |
| 4 | L-Aspartic Acid | C4 H7 N O4 | 133.0373 | 1.495 | 19159 | 56-84-8 |
| 5 | Galactaric Acid | C6 H10 O8 | 210.0377 | 1.522 | 49269 | 526-99-8 |
| 6 | D(-)-Lyxose | C5 H10 O5 | 150.0529 | 1.582 | 78895 | 1114-34-7 |
| 7 | Sucrose | C12 H22 O11 | 342.1164 | 1.623 | 155371 | 57-50-1 |
| 8 | D-(-)-Quinic acid | C7 H12 O6 | 192.0637 | 1.65 | 210012 | 77-95-2 |
| 9 | Fumaric acid | C4 H4 O4 | 116.0108 | 1.833 | 14556 | 110-17-8 |
| 10 | Malic acid | C4 H6 O5 | 134.0217 | 1.86 | 78440 | 6915-15-7 |
| 11 | L-Pyroglutamic acid | C5 H7 N O3 | 129.0427 | 2.24 | 16929 | 98-79-3 |
| 12 | Guanosine | C10 H13 N5 O5 | 283.0919 | 2.365 | 26617 | 118-00-3 |
| 13 | Salidroside | C14 H20 O7 | 300.1209 | 3.727 | 21336 | 10338-51-9 |
| 14 | Ethyl gallate | C9 H10 O5 | 198.0527 | 4.418 | 72958 | 831-61-8 |
| 15 | Protocatechuic acid | C7 H6 O4 | 154.0267 | 5.005 | 68615 | 99-50-3 |
| 16 | Scopolin | C16 H18 O9 | 354.095 | 5.386 | 63868 | 531-44-2 |
| 17 | p-Coumaric acid | C9 H8 O3 | 164.0473 | 6.693 | 22410 | 501-98-4 |
| 18 | p-hydroxylphenylpropanol | C9 H10 O3 | 166.063 | 6.72 | 17538 | 501-97-3 |
| 19 | 4-Hydroxybenzoic acid | C7 H6 O3 | 138.0318 | 6.84 | 133842 | 99-67-7 |
| 20 | 3-O-Caffeoylquinic acid methyl ester | C17 H20 O9 | 368.1106 | 7.847 | 220386 | 123483-19-2 |
| 21 | Aesculin | C15 H16 O9 | 340.0797 | 8.189 | 16172 | 531-75-9 |
| 22 | Caffeic acid | C9 H8 O4 | 180.0421 | 8.243 | 50393 | 331-39-5 |
| 23 | Vitexin -4''-O-glucoside | C27 H30 O15 | 594.1579 | 9.007 | 70281 | 178468-00-3 |
| 24 | Tetrahydrojateorrhizine | C20 H23 N O4 | 341.1627 | 9.197 | 88958 | 13063-54-2 |
| 25 | Narcissoside | C28 H32 O16 | 624.1686 | 9.551 | 28623 | 604-80-8 |
| 26 | Troxerutin | C33 H42 O19 | 742.2314 | 9.659 | 63655 | 7085-55-4 |
| 27 | Verbascoside | C29 H36 O15 | 624.2049 | 10.231 | 43754 | 61276-17-3 |
| 28 | Isoliquiritin apioside | C26 H30 O13 | 550.1685 | 11.959 | 107221 | 120926-46-7 |
| 29 | Isosinensetin | C20 H20 O7 | 372.1208 | 12.066 | 134985 | 17290-70-9 |
| 30 | Isoacteoside | C29 H36 O15 | 624.2053 | 12.571 | 24256 | 61303-13-7 |
| 31 | Naringin | C27 H32 O14 | 580.1793 | 13.442 | 86976 | 10236-47-2 |
| 32 | Narirutin | C27 H32 O14 | 580.1792 | 13.461 | 154054 | 14259-46-2 |
| 33 | Homoplantaginin | C22 H22 O11 | 462.1159 | 14.205 | 25482 | 17680-84-1 |
| 34 | Engeletin | C21 H22 O10 | 434.1209 | 14.422 | 21394 | 572-31-6 |
| 35 | Hesperidin | C28 H34 O15 | 610.1899 | 14.651 | 301492 | 520-26-3 |
| 36 | Tetrahydroberberine | C20 H21 N O4 | 339.1471 | 14.978 | 28178 | 5096-57-1 |
| 37 | Columbianetin | C14 H14 O4 | 246.0894 | 15.483 | 25547 | 3804-70-4 |
| 38 | Formononetin glucoside | C22 H22 O9 | 430.1264 | 16.573 | 21759 | 486-62-4 |
| 39 | Dihydrodaidzein | C15 H12 O4 | 256.0737 | 17.688 | 33128 | 17238-05-0 |
| 40 | Plantainoside D | C29 H36 O16 | 640.1999 | 18.461 | 72642 | 147331-98-4 |
| 41 | Pinobanksin | C15 H12 O5 | 272.0682 | 20.98 | 27251 | 548-82-3 |
| 42 | Ligustroflavone | C33 H40 O18 | 724.2212 | 21.66 | 36926 | 260413-62-5 |
| 43 | Glycyrrhizic acid | C42 H62 O16 | 822.4037 | 25.89 | 239217 | 1405-86-3 |
| 44 | Diammonium glycyrrhizinate | C42 H68 N2 O16 | 822.4031 | 27.386 | 94350 | 79165-06-3 |
| 45 | Sanggenon H | C20 H18 O6 | 354.1102 | 32.246 | 41462 | 86450-80-8 |
| 46 | Magnolol | C18 H18 O2 | 266.1306 | 35.049 | 26181 | 528-43-8 |
| 47 | Honokiol | C18 H18 O2 | 266.1307 | 37.999 | 89286 | 564-73-8 |
| 48 | Effusol | C17 H16 O2 | 252.1147 | 38.396 | 41024 | 73166-28-6 |
